# Low Pneumococcal Vaccination among Patients with Psoriasis in Germany: Results from Vac-Pso

**DOI:** 10.3390/vaccines10071005

**Published:** 2022-06-23

**Authors:** Phoebe Wellmann, Christian Kromer, Ralf Siemer, Selina Klein, Johannes Mohr, Undine Lippert, Andreas Pinter, Dagmar Wilsmann-Theis, Rotraut Mössner

**Affiliations:** 1 Department of Dermatology, University Medical Center Göttingen, 37075 Göttingen, Germany; phoebe.wellmann@stud.uni-goettingen.de (P.W.); christian.kromer@med.uni-goettingen.de (C.K.); johannes.mohr@med.uni-goettingen.de (J.M.); undine.lippert@med.uni-goettingen.de (U.L.); 2 Faculty of Mathematics and Computer Science, University of Göttingen, 37073 Göttingen, Germany; ralf.siemer@stud.uni-goettingen.de; 3 Department of Dermatology and Allergy, University Bonn, 53127 Bonn, Germany; selina.klein@ukbonn.de (S.K.); dagmar.wilsmann-theis@ukbonn.de (D.W.-T.); 4 Department of Dermatology, Venerology and Allergology, University of Frankfurt, 60590 Frankfurt am Main, Germany; andreas.pinter@kgu.de

**Keywords:** influenza, pneumonia, streptococcus pneumoniae, vaccine, psoriasis, atopic dermatitis, pneumococcal infection

## Abstract

While suboptimal pneumococcal vaccination rates have been reported in immunosuppressed patients with rheumatic diseases, data for patients with psoriasis (PsO) or atopic dermatitis (AD) are scarce. Pneumococcal vaccination in Germany is recommended in patients with certain comorbidities, immunosuppression, and/or aged 60 years or above. The aim of this multicenter cross-sectional study was to investigate the pneumococcal vaccination rate in patients with PsO compared to patients with AD and to evaluate patient perceptions. All patients completed a questionnaire on vaccination status and perceptions, patient and disease characteristics, as well as comorbidity. Medical records and vaccination certificates were reviewed. Over the whole cohort (n = 327 PsO (41.9% female), n = 98 AD (42.9% female)), 83.8% and 42.9% of PsO and AD patients, respectively, had an indication for pneumococcal vaccination due to immunosuppressive treatment. The pneumococcal vaccination rate was 14.4% and 10.2% in PsO and AD patients, respectively. The vaccination rate depended significantly on age, working status and presence of psoriatic arthritis. The most common reason for nonvaccination was lacking recommendation by physicians. Higher awareness, particularly for vaccination indication due to immunosuppression among dermatologists, general physicians, and patients, is warranted.

## 1. Introduction

Psoriasis (PsO) is a chronic inflammatory skin disease with a prevalence of approximately 2% in European countries [1] and is associated with a wide range of comorbidities (such as psoriatic arthritis (PsA), obesity, cardiovascular diseases, metabolic disorders, and chronic kidney diseases) [2,3]. According to current guidelines, systemic therapies including conventional immunosuppressive agents, new small molecules, and biologics may be applied in moderate-to-severe PsO [4,5]. Due to comorbid diseases and immunosuppressive/immunomodulatory treatment, PsO patients are, among other patients with chronic inflammatory diseases, prone to a higher risk for infectious diseases such as pneumonia [6,7,8,9]. *Streptococcus pneumoniae* constitutes the most common pathogen-causing bacterial pneumonia [10], but may also cause meningitis, otitis media, sinusitis and sepsis [10,11]. Pneumococcal vaccination reduces the severity of infections and has been reported to lead to lower hospital admission rates and fewer visits to emergency departments in patients with rheumatic diseases [12]. Considering the antipneumococcal immune response of the vaccination, both healthy patients and those with comorbidity showed adequate increase in antibody titers, with adults over 80 years of age exhibiting lower levels (though they still exceed baseline) as van Deursen et al. observed in a study population aged ≥ 65 years [13].

The permanent vaccination commission (STIKO) of the Robert Koch Institute (RKI) recommends pneumococcal vaccination for all persons ≥ 60 years of age, patients treated with immunosuppressants, as well as for patients with certain chronic conditions (congenital or acquired immunodeficiency, chronic heart or lung diseases, metabolic diseases, neurological diseases, and chronic kidney diseases). Depending on the indication, recommendation of the individual vaccine and schedule of vaccination differs (Supplementary Table S1; [14,15]). Healthy adults ≥ 60 years of age are advised to receive the PPSV23 vaccine (Pneumovax^®^; MSD Sharp & Dohme GmbH, Munich, Germany) once; PPSV23 booster vaccinations may be considered every six years. The same schedule is recommended for adults with chronic heart, lung, metabolic, or neurologic diseases, irrespective of their age. Immunocompromised patients should receive a sequential vaccination with PCV13 (Prevenar 13^®^; Pfizer Pharma GmbH, Berlin, Germany) followed by PPSV23 after 6–12 months and PPSV23 booster vaccinations at six-year intervals [14,15]. In patients who had received PPSV23 vaccination prior to immunosuppressive therapy, PCV13 vaccination is recommended after one year [15]. German dermatologic guidelines on vaccination and immunosuppression summarize the STIKO recommendations and their application in dermatologic diseases and treatment [16]. PsO is not mentioned explicitly as indication for pneumococcal vaccination; however, it most probably should satisfy the criterium ‘chronic disease’ for vaccination with PPSV23, but even apart from the presence of psoriasis, for many PsO patients there is an indication for pneumococcal vaccination according to STIKO recommendations in Germany due to other factors such as age, comorbidity and/or immunosuppression [14,15]. In the Unites States of America, PsO is explicitly listed as an indication for vaccination [4].

We previously reported influenza vaccination rates in psoriatic patients in the season 2019/2020 in a German multicenter cohort [17]. While pneumococcal vaccination has been studied extensively in other inflammatory diseases such as rheumatoid arthritis (RA) [18,19], hitherto, the evidence on pneumococcal vaccination rates in PsO patients in Germany is limited. Thus, the aim of this study was to investigate the rate of pneumococcal vaccination in patients with PsO compared to patients with atopic dermatitis (AD) and to evaluate patient perceptions.

## 2. Materials and Methods

### 2.1. Study Design

This multicenter cohort study was conducted in psoriatic patients enrolled at the outpatient and inpatient dermatological clinics of three German university medical centers (Bonn, Frankfurt/Main, and Göttingen). In addition, a smaller cohort of patients with AD was recruited for comparison. Dermatologist-diagnosed PsO or AD, age ≥ 18 years, and the ability to complete the survey were defined as inclusion criteria. All patients gave written informed consent prior to study participation. The study was performed according to the principles of the Declaration of Helsinki [20] and was approved by the Ethics Committees (central ethics committee Göttingen: 40/2/19).

### 2.2. Data Collection

A paper-based questionnaire was used to collect data on sociodemographic characteristics (age, gender and partnership status), occupational status, disease characteristics (disease duration, disease severity (Psoriasis Area and Severity Index (PASI), Eczema Area and Severity Index (EASI)), health-related quality of life (Dermatology Life Quality Index (DLQI)), current and former dermatological therapy, and comorbidity (PsA, atopic, metabolic, psychiatric, and neoplastic diseases, and smoking status) between 08/2019 and 03/2020. Moreover, data on a history of previous infectious diseases (pneumonia, bronchitis, and herpes zoster) was gathered. We reviewed medical records to complement information of the questionnaire (information on comorbidity, course of therapy and disease characteristics). Furthermore, the individual vaccination status regarding pneumococcal vaccination was retrieved from the vaccination certificate, if available, on which analysis vaccination rates was based. For patients who were unable to provide a vaccination certificate, a negative vaccination status was assumed. In addition, study participants provided information in the questionnaire on who recommended and carried out the vaccination, on its tolerability and on the reasons that were decisive in the patient’s choice for or against vaccination. This patient-reported information was based on the number of patients who reported pneumococcal vaccination in the questionnaire. 

### 2.3. Statistical Analysis

Statistical analysis was conducted with opensource software RStudio^®^ (version 3.4.4, Boston, MA, USA), and figures were generated with Prism^®^ (version 9.1.0, GraphPad Software, San Diego, CA, USA). Cohort characteristics and vaccination details were analyzed descriptively. For subgroup analysis, Student’s t-test and Mann–Whitney U test for normally and nonnormally distributed continuous variables as well as Pearson’s χ^2^-test for discrete-valued variables were utilized, respectively. We conducted regression analysis to assess the influence of selected variables including patient characteristics (age, gender, working status), PsA, further comorbidity, immunosuppressive therapy, and history of infectious diseases on the probability of a pneumococcal vaccination in PsO patients. The significance level was set to *p* < 0.05.

## 3. Results

### 3.1. Study Cohort

A total of 327 PsO patients and 98 AD patients participated in the study (Table 1). Detailed patient and disease characteristics of the cohorts have been reported previously [17]. The majority of PsO and AD participants were male (58.1% for PsO and 57.1% for AD). The mean age was 53.4 years for PsO and 44.3 years for AD, respectively. More than half of all patients with PsO (57.2%) suffered from concomitant PsA. In addition, metabolic and psychiatric diseases were found to be common in both PsO and AD. Approximately one third of both AD and PsO patients were active smokers. Regarding infectious diseases, a history of bronchitis was reported by approximately every third patient, and a history of pneumonia was reported by approximately one out of five patients (Table 1).

Disease activity and treatment characteristics of our cohort have been published previously [17]. Briefly, disease activity and impairment of quality of life were low in PsO (median PASI: 1.8, median DLQI: 3.0) and moderate in AD (median EASI: 10.6, median DLQI: 12.0; Supplementary Table S2). At the time of data collection, almost all PsO patients were treated with systemic therapy (92.0%), particularly biologicals (74.0%), while 39.8% of AD patients received systemic treatment (Supplementary Table S2).

### 3.2. Pneumococcal Vaccination Rate

**Figure 1:**
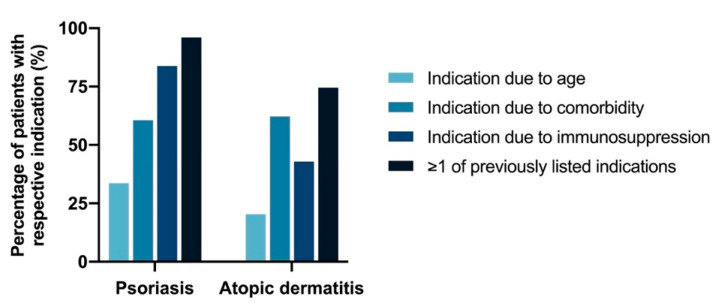
Percentage of patients with indication for pneumococcal vaccination. The figure shows the percentage of PsO and AD patients with respective indications for pneumococcal vaccination according to STIKO recommendation [14]. “≥1 of previously listed indications” depicts all patients that have at least one indication for pneumococcal vaccination. The vast majority of patients (PsO: 96.0% and AD: 74.5%) had at least one indication for vaccination. STIKO: permanent vaccination commission of the Robert Koch Institute.

According to the STIKO recommendations, standard indication for pneumococcal vaccination according to age (≥60 years) applied to 110 of 327 (33.6%) PsO and 20 of 98 (20.4%) AD patients (Figure 1). In younger patients, pneumococcal vaccination was frequently indicated due to comorbidity (54.8% for PsO and 55.1% for AD) and/or immunosuppressive therapy (84.3% for PsO and 43.6% for AD). Across all age groups, pneumococcal vaccination was indicated due to comorbidity in 60.6% and 62.2% and due to immunosuppression in 83.8% and 42.9% of PsO and AD patients, respectively. Thus, the overall indication for pneumococcal vaccination applied to 96.0% of PsO and 74.5% of AD patients (Figure 1). 

**Figure 2:**
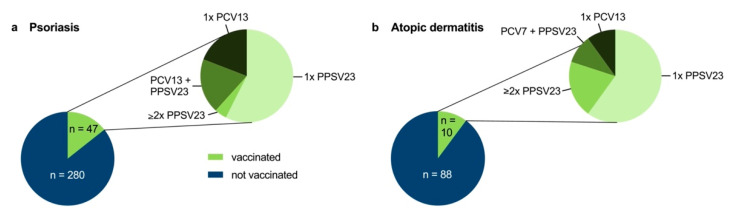
Pneumococcal vaccination rate in PsO and AD patients. PsO (**a**) and AD (**b**) patients with a pneumococcal vaccination at some point according to vaccination certificate were categorized as vaccinated. Detailed breakdown of the different types of pneumococcal vaccines administered is shown in green.

Vaccination certificates could be provided by about two-thirds of patients (69.7% and 70.4% of PsO and AD patients, respectively). Based on the vaccination certificates, a total of 47 of 327 PsO patients (14.4%) and 10 of 98 AD patients (10.2%) had received at least one pneumococcal vaccination at some point (Figure 2). The majority of vaccinated patients had been vaccinated with one dose of PPSV23 (57.4% and 60.0% in PsO and AD, respectively), while sequential vaccination with PPSV23 and PCV7/13 as recommended in immunosuppressed patients according to the STIKO was received by few vaccinated patients (9/47 (19.1%) for PsO and 1/10 (10.0%) for AD).

**Figure 3:**
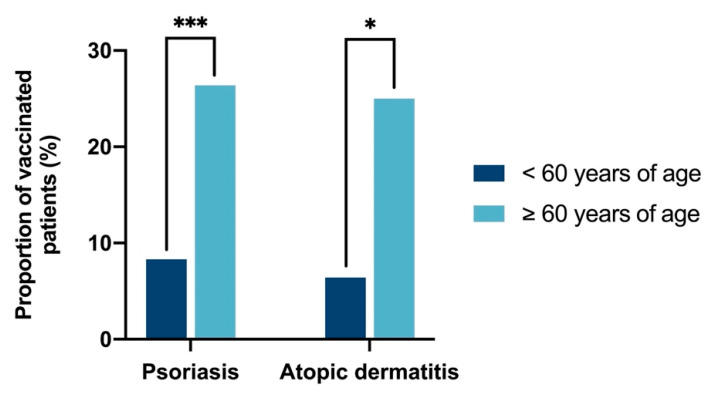
Pneumococcal vaccination according to age. Pneumococcal vaccination rate among the age groups < 60 years of age (PsO: n = 217 and AD: n = 78) and ≥ 60 years of age (PsO: n = 110 and AD n = 20). Patients with a pneumococcal vaccination at some point according to vaccination certificate were categorized as vaccinated. Patients ≥ 60 years of age showed a significantly higher vaccination rate than patients < 60 years of age (26.4% vs. 8.3%, *p* < 0.001 for PsO and 25.0% vs. 6.4%, *p* < 0.028 for AD). * *p* < 0.05; *** *p* < 0.001.

**Figure 4:**
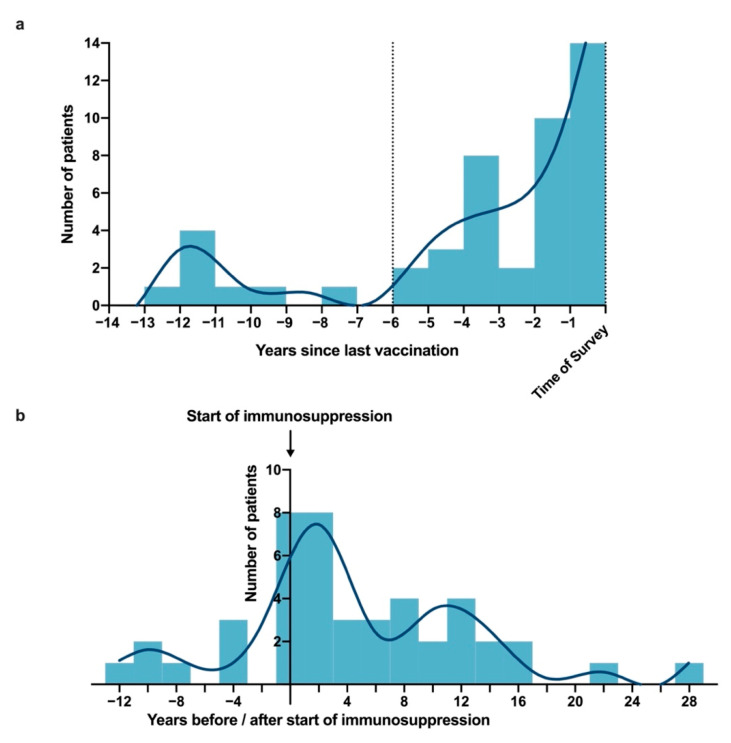
Years since last pneumococcal vaccination and years of pneumococcal vaccination in relation to immunosuppressive therapy. (**a**) Frequency distribution of years since patients’ last pneumococcal vaccinations. A bin width of one year was chosen. The dotted lines enclose all patients vaccinated in the last six years, which is the STIKO recommended time frame after which pneumococcal booster vaccinations may be considered [14]. Patients with a pneumococcal vaccination at some point according to vaccination certificate were categorized as vaccinated (n = 47). (**b**) Frequency distribution of years between the first pneumococcal vaccination and the start of the patient’s immunosuppressive therapy. Only the 45 PsO patients who received immunosuppressive therapy and pneumococcal vaccination at some point were considered in this figure.

Pneumococcal vaccination according to STIKO recommendations (i.e., age, immunosuppression, and booster vaccination) is displayed in Figure 3 and Figure 4. In individuals with PsO aged 60 years and older, the vaccination rate was significantly higher than in younger patients (26.4% vs. 8.3%, *p* < 0.001; Figure 3). Of the 47 PsO patients vaccinated against pneumococci, 39 (83.0%) had received the vaccination within the last 6 years (Figure 4a). At the time of the first pneumococcal vaccination, the mean age of PsO patients was 57.8 years (SD: 9.4). At some point, 45 of the 47 vaccinated PsO patients were treated with immunosuppressive therapy, and 22.2% of those were vaccinated before initiation of the first recorded immunosuppressive therapy (Figure 4b). A total of 13.8% of pneumococcal vaccinations were administered in PsO patients receiving nonbiological immunosuppressive therapy (i.e., cyclosporine, fumaric acid ester, leflunomide, methotrexate, tofacitinib) at that time, while treatment with biologicals was present in 44.8% of PsO patients at time of vaccination. However, due to imprecise documentation of courses of treatment in medical records, the extent of immunosuppressive treatment at the time of vaccination could not be determined accurately in 12.1% of pneumococcal vaccinations.

Supplementary Table S3 shows univariate subgroup analysis with regard to patient and disease characteristics. The age of vaccinated PsO participants was significantly higher than in unvaccinated individuals (62.1 vs. 51.9 years; *p* < 0.001). Among PsO patients, the likelihood of pneumococcal vaccination was also significantly associated with the additional presence of various comorbid diseases in PsO patients (PsA: *p* = 0.001, arterial hypertension: *p* = 0.002, depression: *p* = 0.034, and history of pneumonia: *p* = 0.035) and AD patients (asthma: *p* = 0.016 and postherpetic neuralgia: *p* = 0.039). The rate of patients working full-time was lower in vaccinated compared to unvaccinated PsO patients (*p* < 0.001). Moreover, lower PASI values in PsO patients correlated significantly with a positive vaccination status (*p* = 0.024).

According to multivariate regression analysis for patients with PsO, age ≥ 60 years (*p* < 0.001), not working full-time (*p* = 0.027), and PsA (*p* = 0.014) predicted the likelihood of pneumococcal vaccination significantly (Supplementary Table S4). The variables gender, further comorbidity, history of pneumonia or bronchitis, or immunosuppressive therapy were not significantly associated with the probability of pneumococcal vaccination.

### 3.3. Recommendation and Patient Perspectives

**Figure 5:**
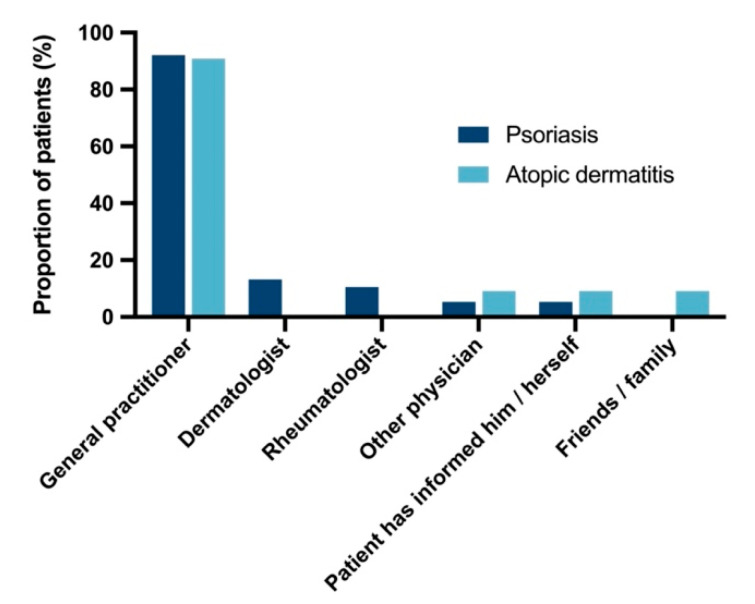
Recommendation of pneumococcal vaccination. The total number of patients according to recommendation of a pneumococcal vaccination by a health care provider or friends/family is depicted. The proportion of patients is based on the patients who had been vaccinated according to the questionnaire (n = 38 for PsO and n = 11 for AD). Multiple indications were permitted.

**Figure 6:**
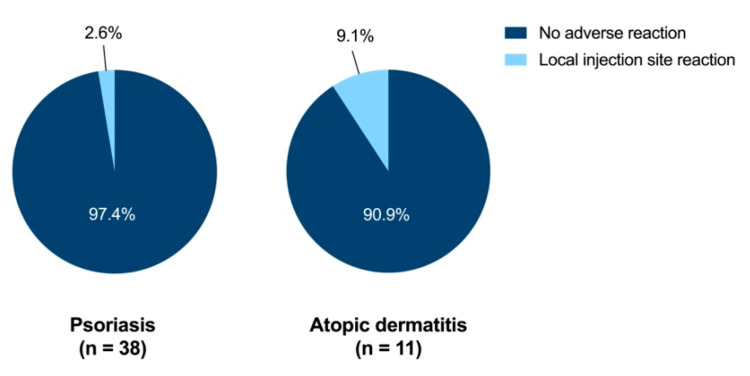
Tolerability of pneumococcal vaccination. Analysis is based on the patients who had been vaccinated according to the questionnaire (n = 38 for PsO and n = 11 for AD). The pie charts display the tolerability of pneumococcal vaccination in psoriasis and atopic dermatitis patients. There were no systemic reactions such as fever, fatigue or exhaustion or other serious health disorders.

Vaccination status differed substantially between documentation in the vaccination certificates (n = 57) and reports in the questionnaire (n = 49) with only n = 33 patients with congruent data. Analysis of recommendation and patient perspectives were based on reported data in the questionnaire. Pneumococcal vaccination was recommended by the general practitioner in most patients who reported pneumococcal vaccination in the questionnaire (35/38 (92.1%) for PsO and 10/11 (90.9%) for AD, Figure 5). The vaccine was administered almost exclusively by general practitioners (94.7% for PsO and 100% for AD). Except for local reactions at the injection site in 2.6% of PsO patients and 9.1% of AD patients, no adverse events were reported (Figure 6). Particularly, no patient indicated systemic symptoms such as “fever, fatigue, or exhaustion” or “other serious health disorder” which were displayed as distinct options in the questionnaire.

Reasons for or against vaccination were comparable among PsO and AD patients according to the vaccinations reported in the questionnaire (Table 2). Approximately two thirds of the patients who reported they had been vaccinated indicated their physicians’ recommendation for vaccination as the reason for vaccination (63.2% for PsO and 63.6% for AD), followed by the general recommendation for vaccination (31.6% for PsO and 27.3% for AD) and presence of comorbidity (21.1% for PsO and 27.3% for AD). The predominant reason against pneumococcal vaccination was a lacking recommendation by a physician in 76.7% and 74.4% of PsO and AD patients who reported they had not been vaccinated, respectively.

## 4. Discussion

To our knowledge, this is the first study to investigate pneumococcal vaccination prevalence in psoriatic patients. Overall, we found a relatively low vaccination rate of 14.4% in PsO and 10.2% in AD, considering that almost all patients had an indication for vaccination due to age, immunosuppressive medication, and/or comorbid diseases.

Vaccination rates have been previously reported in other chronic inflammatory diseases [22,23,24,25]. In a German claims data analysis, the pneumococcal vaccination rate 2013 in RA was investigated, with a comparable vaccination rate of 15.0%, while the vaccination rate in the matched control group without RA was reported to be lower (10.0%) [23]. A Canadian survey on patients with RA found a pneumococcal vaccination rate of 36.7% (36.9% PPSV23, 11.1% PCV13, and 25.0% both) [24]. However, it has to be noted that only patients ≥ 50 years of age were included in that study. The authors identified lacking information about the vaccine (48.4%) and lacking discussion about vaccination with their physician (16.1%) as main reasons against pneumococcal vaccination. Similarly, we found lacking recommendation by a physician to be the main reason against vaccination in our cohort. A Greek study on pneumococcal vaccination prevalence in autoimmune rheumatic disease patients with a similar age distribution to our study (patients aged 18 years and older were included with a comparable proportion of older patients (30.1% > 65 years of age)) reported a high vaccination prevalence rate of 49.37% [25]. In accordance with our study, a lacking recommendation by the caring physician was reported as the main reason against vaccination (82.50%). As was the case in the Greek study’s findings, a regression analysis performed on our PsO patient cohort identified age ≥ 60 years as a significant predictor for a higher probability of a positive vaccination status. Interestingly, neither the presence of further comorbidities (except for PsA) nor intake of immunosuppressant agents had a significant impact on the vaccination status in our cohort. One reason might be that patients with concomitant PsA receive additional counseling by rheumatologists. Theidel and colleagues analyzed the pneumococcal vaccination rate in the general population in Germany according to claims data of a German statutory health insurance of 2008/09 [26]. Of note, they estimated a vaccination prevalence in individuals aged 60 years and above of 50.9% according to a simulation model, while the prevalence in our cohort amounted to 26.4% in the same age group. In comparison, a nationwide study by the STIKO based on German insurance data, which investigated the vaccination prevalence of 60–72-year-olds without risk factors, found a vaccination rate of 24.2% [27]. Overall, a vaccination rate of 19.0% in adult patients with disease-related indication was reported, compared to 14.4% in our cohort. Poethko-Müller and Schmitz identified a vaccination rate of 31.4% for German adults aged 65 to 79, with data gathered from 2008 to 2011 [28]. Thus, in our study, the pneumococcal vaccination rate among patients aged 60 years and older was comparable to the general population in Germany. Due to the finding that in the age group ≥ 60 years of age, the vaccination rate was significantly higher than in younger patients, we hypothesize that there is awareness among physicians for the indication “age”. However, other indications (comorbidity, immunosuppression) seem to be less established in clinical practice. Furthermore, in our cohort, only 19.1% of vaccinated PsO patients received sequential vaccination with PCV13 and PPSV23, which should have been applied in 83.8% of PsO patients due to their immunosuppressive therapy according to STIKO recommendation. Considering the pivotal role of the general practitioner in recommending and performing vaccinations, as indicated in our study, and the dermatologist who prescribes immunosuppressive therapies, a closer interaction would be desirable to offer patients counseling in the decision-making process and ultimately increase vaccination rates. Of note, we found a substantial discrepancy between documentation in the vaccination certificate (if available) and patient reports in the questionnaire which further highlights the need of close cooperation of general practitioner and dermatologist, possibly beyond mere documentation in the vaccination certificate.

Consistent with our finding of lacking physician’s vaccination recommendation being a major determinant of negative vaccination status, Lindsley et al. [29] concluded that vaccination recommendation resulted in increased vaccination rates in patients treated with immunosuppressants due to a dermatological condition. This effect was also identified in other patient populations, for example in diabetic and cardiology patients, indicating that awareness about the beneficial aspects of vaccination brought forward by physicians proves to have a positive influence on vaccination status [30,31]. Informational campaigns by healthcare professionals and providers could address the patient’s fear of contracting pneumonia and establish confidence in the vaccination [32]. Huang et al. have confirmed that a patient’s perceived susceptibility of infection, perceived benefit of vaccine, and recommendation from the government are positively linked to patients’ willingness to receive the vaccination [33]. Government-lead campaigns could therefore provide the general population with important information on the risk of infection as well as safety and benefits of the vaccination. Campaigns addressing the general population could have advantageous effects for not only PsO patients but also for all at-risk patient groups, including, for example, lung cancer patients, in which vaccination rates are also low [34].

Owing to concerns about potentially lower efficacy, vaccination is recommended to be administered prior to initiation of immunosuppressive therapy [14]. Recent studies have shown only slightly reduced immune responses to vaccinations during intake of methotrexate, while the immune response seems not to be attenuated during therapy with other common antipsoriatic drugs [35,36,37,38]. In our study, 22.2% of vaccinated immunosuppressed patients received pneumococcal vaccination before initiation of immunosuppressive treatment. It should also be noted that the vaccine was well tolerated, as only few patients reported local reactions to the injection side, while systemic side effects such as fever or serious health disorders were absent.

The following limitations of our study should be considered. First, vaccination rates were not compared to an age- and sex-matched control group of the general population. In addition, as patients were recruited in specialized tertiary care centers, it can be assumed that there was a bias towards more severely affected patients compared to the general PsO population. Based on their preconceived opinion on vaccinations, some patients may have had selection bias in participating in this study. However, only approximately 10% of patients declined participation in the study. Patients have reported certain information themselves, which puts the given information at risk of imprecision and recall bias. Patients may have been at risk of a time dependent bias when reporting on the tolerability of the vaccination, as patient perception could change over the time span between the last vaccination and the date they filled out the questionnaire. Moreover, the vaccination certificate could not be presented by a substantial number of patients. Furthermore, there might be a discrepancy in the lifetime vaccination prevalence difference between younger and elderly patients, as the prevalence of pneumococcal vaccination increases with age. A major strength of this study is the multicentric, cross-sectional design and the methodology of using both vaccination certificates and patient reports.

In conclusion, pneumococcal vaccination rates in PsO patients were inadequately low considering current STIKO recommendations, particularly in patients younger than 60 years of age. Moreover, a sequential vaccination, as recommended in immunosuppressed patients, was infrequently performed. Physician’s vaccination recommendation represents a considerable factor in the patient’s decision for vaccination. Thus, awareness should be increased among dermatologists, general physicians, and patients in order to optimize the vaccination rates in the target groups. Patients who are on immunosuppressive therapy or who are candidates for immunosuppressive therapy should also be informed about their indication for vaccination and be provided extensive counseling. The impact of the current global COVID-19 pandemic on vaccination of preventable diseases other than COVID-19 in certain risk groups such as PsO remains to be established.

## Figures and Tables

**Table 1:** Cohort Characteristics.

Characteristic	Psoriasis, n (%) ^a^	Atopic Dermatitis, n (%) ^a^
Cohort size	327	98
Female gender	137 (41.9)	42 (42.9)
Age, years, mean (SD)	53.4 (13.9)	44.3 (18.5)
Age at onset of disease, years, mean (SD)	28.9 (16.8)	17.5 (25.0)
Disease duration, years, mean (SD)	24.5 (15.5)	26.8 (18.0)
Family history of PsO	163 (49.8)	21 (21.4)
Family history of AD	50 (15.3)	46 (46.9)
Occupational status		
Working full-time	138 (42.2)	36 (36.7)
Working part-time	38 (11.6)	18 (18.4)
Retired	103 (31.5)	18 (18.4)
**Comorbidity**		
PsA	187 (57.2)	-
Age at onset of PsA, years, mean (SD)	46.1 (13.6)	-
BMI, mean (SD)	29.9 (6.6)	26.0 (5.5)
Obesity (BMI ≥ 30)	142 (43.4)	15 (15.3)
Arterial hypertension	146 (44.6)	30 (30.6)
Cardiovascular disease ^b^	56 (17.1)	12 (12.2)
Dyslipidemia	86 (26.3)	11 (11.2)
Hepatic steatosis	106 (32.4)	7 (7.1)
Hepatic cirrhosis	7 (2.1)	0 (0.0)
Other hepatic diseases ^c^	14 (4.3)	2 (2.0)
Diabetes mellitus	40 (12.2)	4 (4.1)
Chronic kidney disease ^d^	20 (6.3)	4 (5.4)
COPD/emphysema	31 (9.5)	7 (7.1)
Asthma	29 (8.9)	41 (41.8)
Allergic contact dermatitis	28 (8.6)	38 (38.8)
Allergic rhinoconjunctivitis	52 (15.9)	61 (62.2)
Food allergies	27 (8.3)	47 (48.0)
Depression	70 (21.4)	16 (16.3)
Neoplastic diseases ^e^	11 (3.4)	4 (4.1)
**Smoking status**		
Current smoker	112 (34.3)	31 (31.6)
Ex-smoker	144 (44.0)	27 (27.6)
Never smoker	71 (21.7)	40 (40.8)
**Previous infectious diseases**		
Pneumonia	56 (17.1)	18 (18.4)
Years since pneumonia, mean (SD)	15.7 (15.4)	14.8 (8.4)
Hospitalization due to pneumonia ^f^	14 (25.0)	7 (43.8)
Bronchitis	94 (28.7)	29 (29.6)
Years since bronchitis, mean (SD)	7.7 (9.8)	6.2 (8.8)
Hospitalization due to bronchitis ^f^	8 (8.7)	1 (3.6)
Herpes zoster	56 (17.1)	13 (13.3)

^a^ If not indicated otherwise, the number and percentage are depicted. ^b^ Cardiovascular diseases are comprised heart attack, stroke, coronary artery disease, arterial occlusive disease, cardiac insufficiency. ^c^ Other hepatic diseases comprised focal nodular hyperplasia (n = 1), Gilbert’s syndrome (n = 1), hemangioma (n = 2), primary biliary cholangitis (n = 1), hepatitis B (n = 7), hepatitis C (n = 2), and healed hepatitis E (n = 2). ^d^ Chronic kidney disease was assumed if indicated in the questionnaire and, if not indicated in the questionnaire, according to serum creatinine levels using the CKD-EPI (chronic kidney disease epidemiology collaboration) equation 2009 [21]. ^e^ Neoplastic diseases comprised lymphoma (n = 4), breast cancer (n = 4), cervix carcinoma (n = 2), melanoma (n = 2), renal cell carcinoma (n = 1), colon carcinoma (n = 1) and glioblastoma (n = 1). ^f^ The percentage refers to the number of patients with the respective infectious disease (pneumonia, bronchitis, and herpes zoster, respectively). PsO: psoriasis; AD: atopic dermatitis; PsA: psoriatic arthritis; BMI: Body mass index; COPD: chronic obstructive pulmonary disease; SD: standard deviation.

**Table 2:** Reasons for and against pneumococcal vaccination.

	Psoriasis, n (%)	Atopic Dermatitis, n (%)
Number of patients who reported vaccination in the questionnaire	38 (11.6)	11 (11.2)
Reason for vaccination		
Physician’s advice	24 (63.2)	7 (63.6)
General recommendation	12 (31.6)	3 (27.3)
Comorbidity/comedication	8 (21.1)	5 (45.5)
Skin disease	4 (10.5)	0 (0.0)
Treatment of skin disease	7 (18.4)	0 (0.0)
Other reasons ^a^	2 (5.3)	0 (0.0)
**Number of patients who reported no vaccination in the questionnaire**	**289 (88.4)**	**87 (88.8)**
**Reasons against vaccination**		
Lacking recommendation by a physician	198 (68.5)	58 (66.7)
No personal history of severe flu	41 (14.2)	18 (20.7)
Vaccination not deemed necessary by patient	35 (12.1)	11 (12.6)
Lacking confidence in protective effect	11 (3.8)	0 (0.0)
Potential side effects	27 (9.3)	7 (8.0)
Patient forgot/had no time to get vaccinated	33 (11.4)	13 (14.9)
Inflammatory activity of skin disease	4 (1.4)	1 (1.1)
Treatment of skin disease	9 (3.1)	0 (0.0)
Comorbidity/comedication	7 (2.4)	6 (6.9)
Other reasons ^b^	28 (9.7)	9 (10.3)

Analysis was based on the vaccination status as indicated in the questionnaire (n = 38 for PsO and n = 11 for AD).^a^ Other reasons in the psoriasis patient group comprised “patient wanted to prevent pneumonia” (n = 1) and “patient had to be vaccinated according to regulations” (n = 1). ^b^ Other reasons in the psoriasis patient group comprised “advised not to get vaccinated by a physician” (n = 5), “(co)payment” (n = 3), “patient does not want to take too many pharmaceuticals” (n = 2), “vaccination is planned” (n = 3), “patient did not know about the vaccine” (n = 15). Other reasons in the atopic dermatitis patient group comprised “advised not to get vaccinated by a physician” (n = 4), “patient thought the vaccination belonged to the recommended vaccines by the STIKO for children in general” (n = 1), “vaccination is planned” (n = 1), “patient did not know about the vaccine” (n = 2), “patient does not want to be vaccinated before turning 60 years old” (n = 1).

## Data Availability

Not applicable.

## Supplementary Materials

The following supporting information can be downloaded at https://www.mdpi.com/article/10.3390/vaccines10071005/s1, Table S1: Indication for pneumococcal vaccination according to the STIKO; Table S2: Disease characteristics; Table S3: Subgroup analysis with respect to pneumococcal vaccination status; Table S4: Regression analysis predicting pneumococcal vaccination in PsO patients.
