# Supplementary material for: Low Pneumococcal Vaccination among Patients with Psoriasis in Germany: Results from Vac-Pso
Source: Vaccines (Basel). 2022 Jun 23;10(7):1005. doi: 10.3390/vaccines10071005 (PMC9315583; doi:10.3390/vaccines10071005)
Supplement: Supplementary file 1 [file vaccines-10-01005-s001.zip › vaccines-1759613-supplementary.pdf]

vaccines MDPI

Supplementary Material

Table S1. Indication for pneumococcal vaccination according to the STIKO

Indication	Vaccination schedule
Healthy adults **≥ 60 years of age**	PPSV23, booster vaccinations (PPSV23) at intervals of 6 years may be considered
**Chronic diseases** (individuals ≥ 16 years of age)<br>- Chronic cardiovascular diseases or diseases of respiratory system<br>- Metabolic diseases<br>- Neurological diseases	PPSV23, booster vaccination (PPSV23) at intervals of 6 years recommended
Congenital or acquired immunodeficiency (individuals ≥ 2 years of age)<br>- Deficiency or impaired function of T-cells, B-cells, antibodies, complement system, or myeloid cells<br>- (Functional) hyposplenia, asplenia<br>- Neoplastic diseases<br>- HIV infection<br>- Bone marrow transplantation<br>- **Immunosuppressive therapy** (e.g. due to organ transplantation or autoimmune disease)<br>- Chronic kidney failure, nephrotic syndrome, or chronic liver insufficiency	Sequential vaccination with PCV13 and PPSV23 after 6-12 months, booster vaccination (PPSV23) at intervals of 6 years recommended<br><br>In individuals with immunosuppressive therapy, vaccination should be performed before treatment initiation<br><br>In patients who had received PPSV23 vaccination prior to immunosuppressive therapy, PCV13 vaccination is recommended after at least one year after PPSV23 [15].
Diseases and implants with risk of meningitis (individuals ≥ 2 years of age)<br>- Cerebrospinal fluid fistula<br>- Cochlear implant	PPSV23, booster vaccination (PPSV23) at intervals of 6 years recommended

The table shows indications for pneumococcal vaccination and associated vaccination schedules according to the STIKO of RKI [14]. Indications which commonly apply to patients with psoriasis and atopic dermatitis (i.e., age, common comorbid diseases, and immunosuppressive therapy) are indicated in bold. STIKO: permanent vaccination commission of the Robert Koch Institute; HIV: human immunodeficiency virus; RKI: Robert Koch Institute. PPSV23: Pneumovax®; MSD Sharp & Dohme GmbH, Munich, Germany. PCV13: Prevenar 13®; Pfizer Pharma GmbH, Berlin, Germany.

Table S2. Disease characteristics

Characteristic	Psoriasis, n (%)[a]	Atopic dermatitis, n (%)[a]
**Disease activity and quality of life**		
PASI, median (IQR)	1.8 (0.2 - 4.0)	-
EASI, median (IQR)	-	10.6 (2.7-17.2)
DLQI, median (IQR)	3.0 (0.0 - 9.0)	12.0 (5.0 - 18.0)
**Current therapy**		
Phototherapy	1 (0.3)	9 (9.2)
Systemic therapy	301 (92.0)	39 (39.8)
Apremilast	15 (4.6)	-
Cyclosporine	0 (0.0)	8 (8.2)
Fumaric acid ester	17 (5.2)	-
Leflunomide	1 (0.3)	-
Methotrexate	17 (5.2)	1 (1.0)
Tofacitinib	6 (1.8)	-
Retinoids	3 (0.9)	1 (1.0)
Biologics[b]	242 (74.0)	29 (29.6)
TNF-α antagonists	65 (19.9)	-
IL-(12)/23 antagonists	114 (34.9)	-
IL-17(R) antagonists	62 (19.0)	-
Dupilumab	-	29 (29.6)
**Former therapy**		
Phototherapy	152 (46.5)	48 (49.0)
Systemic therapy	287 (87.8)	42 (42.9)
Apremilast	31 (9.5)	-
Corticosteroids	NR	24 (24.5)
Cyclosporine	43 (13.1)	20 (20.4)
Fumaric acid ester	164 (50.2)	-
Leflunomide	15 (4.6)	-
Methotrexate	175 (53.5)	5 (5.1)
Sulfasalazine	12 (3.7)	-
JAK-i[c]	2 (0.6)	-
Retinoids	50 (15.3)	5 (5.1)
Biologics	181 (55.4)	6 (6.1)
TNF-α antagonists	130 (39.8)	-
IL-12/23 antagonists	79 (24.2)	-
IL-17(R) antagonists	55 (16.8)	-
Dupilumab	-	6 (6.1)
Other therapy[d]	19 (5.8)	6 (6.1)
Number of previous therapies, mean (SD)	3.7 (2.6)	3.6 (1.9)

[a] If not indicated otherwise, the number and percentage are depicted. [b] Other biologicals in the PsO group comprised abatacept (n=1). [c] Janus kinase inhibitors (JAK-i) comprised tofacitinib (n=1) and baricitinib (n=1). [d] Other therapy in the PsO group comprised efalizumab (n=12), onercept (n=2), mycophenolatmofetil (n=1), alefacept (n=2), abatacept (n=1), anakinra (n=1), and rituximab (n=1). Other therapy in the AD group comprised autohaemotherapy (n=1), nemolizumab (n=1), and azathioprin (n=1). PASI: Psoriasis Area and Severity Index; EASI: Eczema Area and Severity Index; DLQI: Dermatology Life Quality Index; IQR: interquartile range; NA: not applicable.

Table S3. Subgroup analysis with respect to pneumococcal vaccination status

Characteristic	Psoriasis			Atopic dermatitis		
	**Vaccinated, n (%)[a]**	**Unvaccinated, n (%)[a]**	**p-value [b]**	**Vaccinated, n (%)[a]**	**Unvaccinated, n (%)[a]**	**p-value**
Cohort size	47	280		10	88	
Female gender	25 (53.2)	112 (40.0)	0.110	4 (40.0)	38 (43.2)	1.000
Age, years, mean (SD)	**62.1 (10.4)**	**51.9 (13.8)**	**<0.001**	58.1 (22.3)	42.8 (17.5)	0.061
Age at onset of disease, years, mean (SD)	**34.3 (16.8)**	**27.9 (16.7)**	**0.019**	28.1 (36.6)	16.3 (23.4)	0.340
Disease duration years, mean (SD)	27.8 (17.8)	24.0 (15.0)	0.172	30.0 (29.6)	26.4 (16.4)	0.717
Family history of PsO	18 (38.3)	145 (51.8)	0.114	3 (30.0)	18 (20.5)	0.443
Family history of AD	8 (17.0)	42 (15.0)	0.667	3 (30.0)	43 (48.9)	0.327
**Occupational status**						
Working full-time	**8 (17.0)**	**130 (46.4)**	**<0.001**	2 (20.0)	34 (38.6)	0.317
Working part-time	5 (10.6)	33 (11.8)	1.000	3 (30.0)	15 (17.0)	0.385
**Comorbidity**						
PsA	**37 (78.7)**	**150 (53.6)**	**0.001**	-	-	-
Age at onset of PsA, years, mean (SD)	**51.2 (11.9)**	**44.8 (13.8)**	**0.006**	-	-	
BMI (mean, SD)	31.3 (7.9)	29.7 (6.4)	0.196	25.7 (4.8)	26.0 (5.6)	0.833
Obesity (BMI ≥ 30)	26 (55.3)	116 (41.4)	0.082	1 (10.0)	14 (15.9)	1.000
Arterial hypertension	**31 (66.0)**	**115 (41.1)**	**0.002**	5 (50.0)	25 (28.4)	0.275
Cardiovascular disease[c]	10 (21.3)	46 (16.4)	0.461	2 (20.0)	10 (11.4)	0.353
Dyslipidemia	15 (31.9)	71 (25.4)	0.372	1 (10.0)	10 (11.4)	1.000
Hepatic steatosis	20 (42.6)	86 (30.7)	0.130	1 (10.0)	6 (6.8)	0.541
Hepatic cirrhosis	3 (6.4)	4 (1.4)	0.064	0 (0.0)	0 (0.0)	-
Other hepatic diseases[d]	3 (6.4)	11 (3.9)	0.434	0 (0.0)	2 (2.3)	1.000
Diabetes mellitus	9 (19.1)	31 (11.1)	0.146	0 (0.0)	4 (4.5)	1.000
Chronic kidney disease[e]	3 (6.5)	17 (6.3)	1.000	0 (0.0)	4 (6.2)	1.000
COPD / emphysema	**9 (19.1)**	**22 (7.9)**	**0.027**	2 (20.0)	5 (5.7)	0.149
Asthma	5 (10.6)	24 (8.6)	0.586	**8 (80.0)**	**33 (37.5)**	**0.016**
Allergic contact dermatitis	3 (6.4)	25 (8.9)	0.776	3 (30.0)	35 (39.8)	0.736
Allergic rhinoconjunctivitis	7 (14.9)	45 (16.1)	1.000	5 (50.0)	56 (63.6)	0.496
Food allergies	2 (4.3)	25 (8.9)	0.396	5 (50.0)	42 (47.7)	1.000
Depression	**16 (34.0)**	**54 (19.3)**	**0.034**	3 (30.0)	13 (14.8)	0.207
Neoplastic diseases[f]	2 (4.3)	9 (3.2)	0.663	1 (10.0)	3 (3.4)	0.355
**Smoking status**						
Current smoker	15 (31.9)	97 (34.6)	0.868	2 (20.0)	29 (33.0)	0.497
Ex-smoker	26 (55.3)	118 (42.1)	0.112	4 (40.0)	23 (26.1)	0.456
Never smoker	6 (12.8)	65 (23.2)	0.128	4 (40.0)	36 (40.9)	1.000
**Previous infectious diseases**						
Pneumonia	**14 (29.8)**	**42 (15.0)**	**0.035**	3 (30.0)	15 (17.0)	0.382
Years since pneumonia, mean (SD)	14.0 (16.2)	16.2 (15.3)	0.658	11.3 (8.1)	15.5 (8.6)	0.481

Hospitalization due to pneumonia[g]	4 (28.6%)	10 (23.8%)	0.732	1 (33.3%)	6 (40.0%)	1.000
Bronchitis	17 (36.2)	77 (27.5)	0.290	4 (40.0)	25 (28.4)	0.737
Years since bronchitis	6.2 (6.9)	8.0 (10.4)	0.373	9.3 (11.3)	5.6 (8.5)	0.575
Hospitalization due to bronchitis[g]	0 (0.0)	8 (10.4)	0.342	1 (25.0)	0 (0.0)	0.143
Herpes zoster	12 (25.5)	44 (15.7)	0.146	3 (30.0)	10 (11.4)	0.143
Years since Herpes zoster, mean (SD)	11.8 (13.4)	15.7 (13.4)	0.378	23.0 (16.1)	10.9 (11.4)	0.322
(Post)herpetic neuralgia[g]	10 (83.3)	33 (75.0)	1.000	**1 (33.3)**	**10 (100.0)**	**0.039**
**Disease activity**						
PASI median (IQR)	**2.1 (3.4)**	**1.6 (3.9)**	**0.024**	-	-	-
EASI median (IQR)	-	-	-	7.0 (11.0)	10.8 (14.2)	0.054
DLQI median (IQR)	3.0 (7.0)	3.0 (9.0)	0.771	6.0 (6.3)	13.0 (13.0)	0.112

[a] If not indicated otherwise, the number and percentage are depicted. [b] Differences comparing the subgroups of vaccinated and not vaccinated patients separately for PsO and AD were calculated with Pearson's χ^2-test for discrete-valued variables and student's t-test and Mann-Whitney u test for normally and not-normally distributed continuous variables, respectively. Significant results are printed in bold. [c] Cardiovascular diseases comprised heart attack, stroke, coronary artery disease, arterial occlusive disease, cardiac insufficiency. [d] Other hepatic diseases comprised focal nodular hyperplasia (n=1), Gilbert's syndrome (n=1), hemangioma (n=2), primary biliary cholangitis (n=1), hepatitis B (n=7), hepatitis C (n=2), and healed hepatitis E (n=2). [e] Chronic kidney disease was assumed if indicated in the questionnaire and, if not indicated in the questionnaire, according to serum creatinine levels using the CKD-EPI equation 2009 [38]. [f] Neoplastic diseases comprised lymphoma (n=4), breast cancer (n=4), cervix carcinoma (n=2), melanoma (n=2), renal cell carcinoma (n=1), colon carcinoma (n=1) and glioblastoma (n=1). [g] The percentage refers to the number of patients with the respective infectious disease (pneumonia, bronchitis, and herpes zoster, respectively).

Table S4. Regression analysis predicting pneumococcal vaccination in PsO patients

Variable	Coefficient (SE)	p-value
**Age ≥ 60 years**	**0.004 (0.001)**	**<0.001**
Female[a]	0.029 (0.042)	0.482
**Working full-time[b]**	**-0.079 (0.036)**	**0.027**
**PsA**	**0.091 (0.037)**	**0.014**
Further comorbidity	0.021 (0.021)	0.332
History of pneumonia or bronchitis	0.017 (0.040)	0.680
Immunosuppressive therapy [c]	0.043 (0.048)	0.365
**Constant**	**-0.189 (0.084)**	**0.025**

The dependent variable for the regression analysis was the probability of a pneumococcal vaccination. Independent variables included age, gender, occupational status, PsA, at least one further comorbidity (i.e., asthma, COPD, cardiovascular disease, hepatic cirrhosis, diabetes mellitus, obesity, chronic kidney disease, and neoplastic disease), a history of bronchitis and pneumonia, and immunosuppressive therapy. Significant predictors are printed in bold. [a] The reference category to female was male. [b] The reference category of working full-time was any other occupational status. [c] Just current immunomodulatory or immunosuppressive systemic therapy administered for PsO and PsA were taken into consideration (i.e., fumaric acid ester, methotrexate, tofacitinib, cyclosporine, leflunomide, and biologicals). The model was adjusted for the center of recruitment (not significant). SE: standard error.
